# Cerebrospinal Fluid Biomarkers in Creutzfeldt–Jakob Disease: Diagnostic Value, Limitations, and Future Multi-Omics Strategies

**DOI:** 10.3390/ijms27010553

**Published:** 2026-01-05

**Authors:** Rui Xu, Cao Chen, Qi Shi, Xiao-Ping Dong

**Affiliations:** 1 National Key Laboratory of Intelligent Tracking and Forecasting for Infectious Diseases, NHC Key Laboratory for Medical Virology and Viral Diseases, Beijing Key Laboratory of Viral Infectious Diseases, National Institute for Viral Disease Control and Prevention, Chinese Center for Disease Control and Prevention, Beijing 102206, China; 2 Collaborative Innovation Center for Diagnosis and Treatment of Infectious Diseases, Zhejiang University, Hangzhou 100084, China; 3 Center for Biosafety Mega-Science, Chinese Academy of Sciences, Wuhan 430071, China; 4 China Academy of Chinese Medical Sciences, Beijing 100700, China; 5 Shanghai Institute of Infectious Disease and Biosafety, Shanghai 200003, China

**Keywords:** Creutzfeldt–Jakob disease, cerebrospinal fluid biomarkers, diagnosis, multi-omics strategies

## Abstract

Creutzfeldt–Jakob disease (CJD) is a rare but devastating neurodegenerative disorder characterized by the pathological misfolding of the cellular prion protein (PrP^C^) into the pathogenic isoform-scrapie prion protein (PrP^Sc^), ultimately leading to fatal outcomes. Cerebrospinal fluid (CSF) biomarkers play a pivotal role in early diagnosis, longitudinal monitoring, and prognostic assessment, thereby enhancing the clinical management of this challenging disease. This review summarizes the established CSF biomarkers, 14-3-3 protein, tau protein (total tau), phosphorylated tau isoforms, α-synuclein, neurofilament light chain (Nfl), S100B, neuron-specific enolase (NSE), and phosphorylated neurofilament heavy chain (pNFH), highlighting typical sensitivity ranges (14-3-3 ~70–85%; RT-QuIC > 90%) and subtype-dependent performance variation. We further dissect limitations related to assay variability, inter-laboratory cut-off inconsistencies, and reduced specificity in non-prion dementias. Looking ahead, we discuss emerging multi-omics discovery, integration of CSF with blood-based biomarkers and imaging signatures, and AI-enabled diagnostic modeling. We propose a three-tier biomarker framework combining Real-Time Quaking-Induced Conversion (RT-QuIC) as a confirmatory assay, tau/NfL/pNFH as injury-severity indicators, and multi-omics-derived signatures for early detection and prognosis stratification.

## 1. Introduction

CJD is a rare neurodegenerative disorder causing rapid cognitive decline and behavioral changes, mainly due to misfolded prion proteins that damage the brain. Early diagnosis is crucial due to the disease’s fatal nature and lack of treatments, but traditional methods often fail due to nonspecific symptoms. Recent years, CSF-based biomarkers, including 14-3-3 protein, total tau (t-tau) and amplification assays such as RT-QuIC assay, have markedly improved diagnostic accessibility and timeliness. However, variability in biomarker expression across disease subtypes, lack of assay standardization, and overlapping elevations in non-prion disorders continue to limit clinical certainty [1]. This review aims to evaluate current CSF biomarkers advances in CJD, critically analyze diagnostic performance and limitations, and outline future multi-omics and AI-driven strategies that may support earlier detection, increased accuracy, and individualized prognosis assessment.

## 2. Pathophysiological Basis of CJD

### 2.1. Mechanism of PrP Misfolding

The pathophysiology of CJD is fundamentally linked to the misfolding of the prion protein (PrP), specifically the conversion of the PrP^C^ into the PrP^Sc^. This conformational change is characterized by a shift from an alpha-helical structure to a beta-sheet-rich structure, which is crucial for the protein’s infectious properties. The accumulation of PrP^Sc^ in the central nervous system (CNS) leads to neurodegeneration and is marked by the formation of amyloid plaques and spongiform changes in brain tissue. The propagation of PrP^Sc^ occurs through a self-replicating mechanism where PrP^Sc^ induces the misfolding of neighboring PrP^C^ molecules, thereby amplifying the pathological form. This process is not only confined to localized areas but can also spread trans-synaptically, affecting various brain regions over time. The neurotoxic effects of PrP^Sc^ are attributed to its ability to disrupt cellular functions, leading to neuronal loss and gliosis. Importantly, the unique transmissibility of prion diseases, including CJD, raises significant public health concerns, particularly regarding potential iatrogenic transmission through medical procedures and contaminated biological materials [2,3,4].

### 2.2. Clinical Subtypes and Pathological Signatures

CJD presents with a diverse clinical spectrum, which can be classified into sporadic, genetic, and acquired forms. The sporadic form, accounting for approximately 85% of cases, typically manifests without any known genetic predisposition or external cause. In contrast, genetic forms of CJD are linked to mutations in the *PRNP* gene, leading to distinct clinical and pathological features. The most common subtypes of sporadic CJD (sCJD) include MM1, MV2, and VV2, and mixed phenotypes, which correlate with *PRNP* codon 129 genotype and PrP^Sc^ type. MM1 typically exhibits cortical predominance with rapidly progressive dementia, whereas MV2 subtype may involve deeper nuclei such as the thalamus. These subtype-linked pathological patterns guide interpretation of CSF biomarkers, as tau, α-synuclein, and RT-QuIC positivity may vary with underlying strain biology. Recent integration of neuropathology with CSF biomarkers has improved pre-mortem subtype recognition, enabling more stratified prognosis estimation [5,6,7].

## 3. Importance of CSF Biomarkers in Diagnosis

### 3.1. Advantages and Diagnostic Constraints

CSF biomarkers play an important role in the diagnosis of CJD, particularly given the challenges associated with its clinical diagnosis, which often relies on post-mortem examination. The convenience and repeatability of CSF testing present significant advantages over invasive procedures like brain biopsies and imaging studies. CSF collection via lumbar puncture is a relatively straightforward procedure that can be performed safely and repeatedly, allowing for longitudinal monitoring of biomarker levels as the disease progresses [8]. Furthermore, the concentration of specific biomarkers in CSF, such as total tau (t-tau) and 14-3-3 protein, has been correlated with disease progression, providing valuable insights into the pathophysiological mechanisms underlying CJD [9]. However, the limitations of CSF biomarkers cannot be overlooked. The specificity of certain biomarkers can be compromised due to their elevation in other neurodegenerative conditions, which may lead to misdiagnosis [10]. Additionally, the variability in biomarker levels due to pre-analytical factors, such as the timing of sample collection and processing conditions, poses challenges for standardization and interpretation [11]. Thus, while CSF biomarkers offer a promising avenue for CJD diagnosis, their application must be approached with caution, emphasizing the need for comprehensive diagnostic frameworks that integrate clinical, imaging, and biomarker data [12].

### 3.2. Standardization Challenges

The standardization of CSF collection and processing techniques is crucial for ensuring the reliability of biomarker measurements in the context of CJD diagnosis. One significant factor influencing the stability of CSF biomarkers is the technique used for lumbar puncture. Variations in the procedure can affect the quality and quantity of the CSF sample obtained, potentially leading to erroneous biomarker levels. For instance, the use of different needle sizes, the speed of collection, and the position of the patient during the procedure can all impact the final sample quality [13]. Moreover, the conditions under which CSF samples are stored and transported are critical. Research has shown that biomarkers can degrade or change concentration when subjected to inappropriate storage conditions, such as fluctuations in temperature or repeated freeze–thaw cycles [14]. Therefore, establishing standardized protocols for CSF collection, including strict guidelines for sample handling and processing, is essential to minimize variability and enhance the reproducibility of biomarker analyses. This standardization will not only improve diagnostic accuracy but also facilitate multicenter studies and enhance the comparability of research findings across different laboratories [15]. As the field progresses, ongoing efforts to refine these protocols will be vital in advancing the utility of CSF biomarkers in the clinical setting for CJD and other neurodegenerative diseases.

## 4. Multi-Omics Approaches in Biomarker Discovery for CJD

Rapid advances in multi-omics screening have expanded the biomarker landscape beyond classical neuronal-injury markers. Transcriptomics contributes to disease subtyping and early diagnosis. Tarozzi et al. conducted an integrated analysis of transcriptomic data from sCJD MM1 and VV2 subtypes and found that the VV2 subtype exhibited significant impairments in dopamine secretion, calcium release regulation, and GABA signaling pathways, whereas the MM1 subtype displayed gene expression profiles shared with other neurodegenerative diseases [16]. Additionally, ADAR-mediated RNA editing events were found to be significantly increased in brain samples from CJD patients, a phenomenon that may reflect processes of neuroinflammation and proteostasis dysregulation [16]. Therefore, dynamic monitoring of RNA editing events holds promise as a novel strategy for the early diagnosis of CJD.

Proteomics is the scientific study of the expression patterns and alterations of all proteins within an organism or cell. In CJD research, proteomics has been employed to analyze protein changes in CSF, blood, and other bodily fluids to identify potential biomarkers for early diagnosis. For instance, Saima Zafar et al. [17] utilized RT-QuIC combined with SWATH-MS proteomics to analyze brain tissue samples from mouse models infected with two human prion strains, CJD-MM1 and VV2. Their study revealed multiple proteins, such as Gnl1 and Stxbp1, that were significantly altered during the early stages of the disease, suggesting their potential as candidate biomarkers for early diagnosis. Metabolomics enables the determination of infection stages. Barberini et al. observed significant changes in certain lipid and amino acid metabolites in the blood or CSF of CJD patients, indicating that these alterations may be associated with prion-induced cytotoxicity [18]. Detecting changes in metabolic profiles can reflect the initial stages of the disease. Although multi-omics evidence remains preliminary, the convergence of transcriptomic, proteomic, and metabolic disturbances highlights an evolving opportunity for biomarker expansion and for identifying biological mechanisms underlying strain-specific progression.

Despite these advances, multi-omics technologies in CJD research face several technical and translational bottlenecks. In transcriptomics, particularly for RNA editing analysis, cross-platform variability, sequencing depth requirements, and the lack of standardized bioinformatic pipelines remain major challenges, limiting reproducibility across cohorts. In proteomics, differences in sample preparation, mass spectrometry platforms, and peptide quantification strategies contribute to substantial inter-laboratory variability. Metabolomics is especially sensitive to pre-analytical factors, including sample stability, storage temperature, and batch effects, which can significantly alter metabolite profiles. Therefore, multi-center validation cohorts, standardized operating procedures for sample handling, harmonized data-processing workflows, and orthogonal verification strategies are essential before multi-omics-derived biomarkers can be translated into clinical practice [15,19].

## 5. Major Established CSF Biomarkers for Diagnosis

The protein changes that distinguish CJD from other neurodegenerative diseases are best observed in CSF, as this biofluid directly reflects CNS pathology and is the most reliable source for identifying CJD-specific proteomic biomarkers. The diagnostic performance and clinical utility of major CSF biomarkers (14-3-3, tau protein, prion protein-related biomarkers) in CJD are summarized in Table 1. While Western blot (WB) has historically been used in biomarker research, current clinical and translational practice increasingly relies on quantitative immunoassays and amplification-based techniques for routine CSF biomarker detection and standardization.

### 5.1. 14-3-3 Protein

14-3-3 protein is one of the earliest surrogate CSF biomarkers incorporated into diagnostic criteria for suspected sCJD. The WHO included a positive CSF 14-3-3 result in the clinical criteria for sCJD diagnosis in 1998. The release mechanism of 14-3-3 proteins into the CSF occurs when neuronal cells undergo apoptosis or necrosis, leading to the leakage of intracellular proteins into the extracellular space. This phenomenon is particularly pronounced in neurodegenerative conditions like CJD, where rapid neuronal loss is a hallmark. Studies have demonstrated that elevated levels of 14-3-3 in CSF correlate with the presence of prion diseases, making it a crucial marker for differential diagnosis. However, the sensitivity and specificity of 14-3-3 protein vary across different CJD subtypes. For instance, sensitivity rates can reach 70–85% in sCJD cases, but specificity remains a concern, particularly as elevated levels can also be observed in other neurodegenerative diseases, such as Alzheimer’s disease (AD) and frontotemporal dementia [12,13]. Accordingly, 14-3-3 is best applied as a supportive rather than confirmatory test, and interpretation must be contextualized with magnetic resonance imaging (MRI), diffusion weighted imaging (DWI) hyperintensity, RT-QuIC results, and clinical course.

### 5.2. Tau Protein

Tau protein represents another critical class of biomarkers in the diagnosis of CJD. Tau is a microtubule-associated protein primarily involved in regulating the stability of axonal microtubules. Under pathological conditions, tau may undergo abnormal phosphorylation and aggregate into neurofibrillary tangles, which represent a core feature of several neurodegenerative disorders [20]. In CJD patients, CSF levels of t-tau are significantly elevated, reflecting widespread neuronal damage [21]. In sCJD, elevated t-tau levels demonstrate high diagnostic accuracy, with an area under curve (AUC) of 0.942 when distinguishing it from rapidly progressive AD [22]. Additionally, tau phosphorylated at specific sites (e.g., p-tau181, p-tau217, and p-tau231) shows unique advantages in discriminating CJD from other dementias [23,24].

p-tau181, one of the earliest and most extensively studied phosphorylated forms of tau, exhibits a complex pattern in CJD. Several studies indicate that although CSF t-tau is markedly increased in CJD, p-tau181 levels are generally low—a profile that contrasts sharply with that observed in AD [23]. Moreover, the p-tau181/t-tau ratio has proven effective in distinguishing CJD from AD, particularly in cases of rapidly progressive dementia (RPD) [25]. p-tau217 is considered a more sensitive biomarker for AD than p-tau181, though its role in CJD remains less explored. One study reported significantly elevated CSF p-tau217 levels in CJD patients, which correlated strongly with amyloid pathology [23]. Furthermore, p-tau217 exhibits a wider dynamic range, potentially allowing better reflection of disease progression [26]. Thus, p-tau217 represents a promising candidate biomarker for future CJD diagnostics. Research on p-tau231 in CJD is limited, yet preliminary data suggest diagnostic potential in certain CJD subtypes. For instance, one study found that CSF p-tau231 levels in CJD patients were significantly lower than in AD patients but higher than in healthy controls [27], indicating its possible utility in discriminating between CJD and AD.

Relying solely on either t-tau or a single form of p-tau as a diagnostic marker for CJD may have limitations. Combining p-tau/t-tau ratios can significantly enhance diagnostic accuracy. Notably, the t-tau/p-tau ratio has emerged as a valuable tool for distinguishing CJD from other rapidly progressive neurodegenerative diseases. For example, one study demonstrated that a p-tau/t-tau ratio below 0.075 offers high sensitivity and specificity for CJD diagnosis [28]. The clinical utility of this biomarker is further underscored by its ability to provide insights into underlying pathophysiological mechanisms, linking tau pathology to synaptic dysfunction and neuronal loss [29].

### 5.3. Prion Protein-Dependent Biomarkers

The detection of abnormal PrP^Sc^ in CSF is pivotal for diagnosing prion diseases such as CJD. Among the advanced methodologies, the RT-QuIC assay has emerged as a highly sensitive and specific diagnostic tool. This assay exploits the unique properties of PrP^Sc^ to induce a conformational change in PrP^C^, leading to the amplification of PrP^Sc^ aggregates. The RT-QuIC technique has demonstrated a sensitivity of 90–95% and specificity exceeding 95% for diagnosing sCJD, making it a formidable asset in clinical settings where rapid and accurate diagnosis is crucial [30]. Furthermore, recent discoveries of novel PrP^Sc^ oligomers have underscored their clinical significance. These oligomers, which may represent early pathological changes, have been linked to the onset of neurodegeneration and cognitive decline. Their presence in CSF could provide insights into the disease process and potentially serve as early biomarkers for prion diseases [31]. The implications of these findings are profound, as they not only enhance diagnostic accuracy but also open avenues for therapeutic interventions targeting these oligomeric forms, potentially altering the disease course.

## 6. Other Emerging CSF Biomarkers

### 6.1. Neurofilament Light Chain (NfL)

NfL has emerged as a promising biomarker for axonal injury in various neurodegenerative diseases, including CJD. NfL serves as a pathological marker reflecting neuronal damage and has been shown to correlate with clinical severity and disease progression. The pathophysiological basis role for NfL as a biomarker lies in its release into the CSF following axonal injury, making it a sensitive indicator of neurodegeneration. Recent studies have highlighted NfL’s potential in early diagnosis and disease monitoring in CJD, with elevated levels detected even in the early stages of the disease. This biomarker’s utility is further enhanced by its ability to provide prognostic information regarding disease progression and patient outcomes [12,32]. The ability to measure NfL levels in both CSF and serum using advanced immunoassays allows for broader application in clinical settings, facilitating timely diagnosis and management of CJD. As research continues to elucidate the role of NfL in CJD and other neurodegenerative disorders, it is poised to become a cornerstone in the diagnostic landscape for prion diseases.

In addition to cerebrospinal fluid, recent studies have highlighted the clinical utility of plasma NfL as a minimally invasive biomarker in prion diseases. Plasma NfL levels show a strong correlation with CSF concentrations and reflect the extent of axonal damage and disease progression. Although plasma NfL lacks disease specificity and cannot replace prion-specific assays, it may serve as a useful complementary tool for initial screening, longitudinal monitoring, and follow-up in settings where lumbar puncture is not readily feasible [33].

### 6.2. Inflammation-Related Biomarkers

CSF biomarkers associated with inflammation have gained attention in understanding the pathophysiology of prion diseases, particularly in relation to neuroinflammation observed in CJD. Cytokines such as interleukin-6 (IL-6) and tumor necrosis factor-alpha (TNF-α) have been implicated in the inflammatory response during CJD progression. Elevated levels of these cytokines in CSF correlate with the degree of neuroinflammation and neuronal damage, suggesting their potential role as biomarkers for disease severity and progression [34]. However, available evidence indicates that inflammation-related biomarkers are better suited as adjunct rather than stand-alone diagnostic markers. Multicenter cohort studies suggest that although CSF IL-6 and TNF-α levels correlate with disease severity and rapid progression, their diagnostic specificity is limited due to elevations in other neuroinflammatory and infectious conditions. Accordingly, current data support their use primarily for monitoring neuroinflammatory activity and disease dynamics, rather than for primary differential diagnosis [34]. Additionally, the complement system’s activation has been explored as a potential indicator of neuroinflammatory processes in CJD. The presence of complement activation products in CSF may reflect ongoing inflammatory responses and could serve as a valuable tool for monitoring disease activity. The clinical utility of these inflammation-related biomarkers lies in their ability to provide insights into the underlying mechanisms of neurodegeneration and to guide therapeutic strategies aimed at modulating the inflammatory response. As research continues to elucidate the complex interplay between inflammation and neurodegeneration in prion diseases, these biomarkers may become integral to both diagnostic and prognostic assessments, ultimately improving patient management and outcomes [35]. In addition, multiplex bead-based immunoassays, such as Luminex platforms, have been increasingly applied in research settings to enable simultaneous quantification of multiple CSF biomarkers, particularly for exploratory analyses of inflammatory mediators and synaptic injury markers. However, their use remains primarily complementary, as assay standardization and cross-platform comparability are still under evaluation.

### 6.3. α-Synuclein

α-synuclein is a soluble protein predominantly expressed in the central nervous system, with its abnormal aggregation constituting a core pathological feature of synucleinopathies such as Parkinson’s disease (PD) and dementia with Lewy bodies (DLB) [36]. Studies indicate that α-synuclein not only participates in synaptic function regulation but may also promote neurodegenerative pathology through mechanisms of cell-to-cell propagation [37]. In prion diseases, accumulating evidence suggests that α-synuclein is not merely a passive marker of neuronal injury, but may participate in prion protein-mediated neurodegeneration. Experimental studies have demonstrated that misfolded prion protein can facilitate α-synuclein aggregation, intracellular trafficking, and synaptic dysfunction, thereby amplifying neurotoxic signaling cascades and accelerating neuronal loss. This prion–synuclein interaction provides a plausible biological explanation for the marked elevation of CSF α-synuclein observed in specific sCJD subtypes, particularly MM1, and supports its role as a surrogate indicator of rapid synaptic and axonal degeneration [37,38].

Across multiple studies, CSF α-synuclein has demonstrated high sensitivity and specificity in distinguishing prion disease patients from non-prion disease controls. For example, a meta-analysis showed that CSF α-synuclein can discriminate prion disease from other neurodegenerative diseases with 92% sensitivity and 96% specificity [39]. Nevertheless, its diagnostic performance remains inferior to certain classical biomarkers, such as total tau and 14-3-3 protein. Nonetheless, a unique advantage of α-synuclein lies in its distribution pattern, which can reflect subtype-specific features of prion diseases. For instance, in sCJD MM1 subtype, CSF α-synuclein levels are markedly higher than in other subtypes [40]. Beyond diagnostic value, CSF α-synuclein also demonstrates strong prognostic potential. Mastrangelo et al. reported an inverse relationship between CSF α-synuclein levels and survival time in prion disease, particularly in the most common sCJD subtype [41]. Similarly, Abu-Rumeileh et al. corroborated this association and noted that CSF β-synuclein exhibits a comparable prognostic capability [42]. Moreover, fluctuations in CSF α-synuclein may reflect disease dynamics: in sCJD patients, levels increase as disease worsens, consistent with its role as a marker of neuronal injury [38].

### 6.4. S100B

S100B is a calcium-binding protein predominantly secreted by astrocytes and plays a critical role in maintaining CNS homeostasis [43]. However, under pathological conditions such as traumatic brain injury (TBI), stroke, or neurodegenerative diseases, S100B is released from the injured CNS into the bloodstream and cerebrospinal fluid, thereby serving as an important biomarker of neural injury. Evidence indicates that elevated S100B levels not only reflect the extent of acute neural injury but also predict patients’ clinical outcomes and treatment responses [43]. From a mechanistic perspective, elevated S100B levels in CJD are thought to reflect astrocyte activation and calcium-dependent inflammatory signaling. Recent studies further suggest that host genetic background, particularly *PRNP* codon 129 polymorphism, may modulate astroglial responses to prion pathology. This interaction provides a biologically plausible explanation for the diagnostic value of S100B in certain genetic and atypical prion disease subtypes, especially in cases where classical prion-specific assays such as RT-QuIC are negative [44].

In certain contexts, S100B, demonstrates higher diagnostic potential. For example, Sanchez-Juan et al. reported in a study on sCJD that the sensitivity of S100B is comparable to that of 14-3-3 and tau, and in some cases even higher [45]. Moreover, changes in S100B levels are closely related to patient age, disease duration, and the polymorphism at codon 129 of the *PRNP* gene [45]. Notably, the diagnostic value of S100B is not limited to sCJD. In genetic CJD (gCJD), S100B also shows significant elevations. For instance, in patients carrying the *PRNP* E146G mutation, CSF S100B levels exceed twice the upper limit of normal, while other markers such as 14-3-3 and RT-QuIC are negative [44]. This finding suggests that S100B may play a distinctive role in differentiating among different types of prion diseases.

### 6.5. Neuron-Specific Enolase (NSE)

NSE is a glycolytic enzyme primarily found in neurons and neuroendocrine cells. When these cells are damaged or die, NSE is released into the CSF and blood [46]. Therefore, elevated levels of NSE in CSF can serve as a sensitive indicator of neuronal injury. Multiple studies have shown that in patients with CJD, the levels of NSE in CSF are significantly increased. For example, one study found that in the early stages of the disease, the CSF NSE levels in CJD patients were much higher than those in normal controls and patients with other neurological diseases [47]. Additionally, Kropp et al. analyzed longitudinal CSF samples from 16 CJD patients and found that NSE levels continued to rise during the course of the disease, with this change being associated with disease progression [46]. Although NSE demonstrates high sensitivity in the diagnosis of CJD, its specificity is relatively low. Zerr et al. reported that when the CSF NSE concentration exceeds 35 ng/mL, the optimal sensitivity for diagnosing CJD is 80%, with a specificity of 92% [48]. However, another study indicated that when used alone as a diagnostic criterion, the specificity of NSE is only 91.5% [49].

### 6.6. Phosphorylated Neurofilament Heavy Chain (pNFH)

pNFH is an important member of the neurofilament protein family, primarily distributed in the axons of the central nervous system. When axonal injury or neuronal death occurs, pNFH is released from the damaged axons into the CSF and blood, thus its levels can reflect the degree of neuronal injury [33,50]. Studies have shown that pNFH is significantly elevated in various neurodegenerative diseases (such as AD, PD, amyotrophic lateral sclerosis, etc.), but multiple studies have indicated that the CSF levels of pNFH in CJD patients are significantly higher than those in other neurodegenerative diseases and healthy controls [51]. Compared with the traditional 14-3-3 protein, pNFH has higher sensitivity and specificity. One study found that the AUC of pNFH reaches above 0.9, indicating its strong diagnostic ability in distinguishing CJD from other diseases [33]. Moreover, the changes in pNFH levels are closely related to the severity and progression rate of the disease, which provides the possibility for it to serve as a dynamic monitoring indicator. Compared with the 14-3-3 protein, pNFH shows better diagnostic performance, especially in young patients [33]. In addition, serum NfL, as a non-invasive detection method, also shows similar diagnostic value to pNFH, but its specificity is slightly lower than that of pNFH, especially when distinguishing CJD from other rapidly progressive dementias [51,52]. pNFH can not only be used for the initial diagnosis of the disease, but also as a dynamic monitoring indicator for disease progression. Studies have shown that as the disease progresses, pNFH levels gradually increase and may decrease after therapeutic intervention [33]. This characteristic endows pNFH with potential application value in evaluating treatment effects and predicting disease prognosis. However, the detection methods and reference ranges used in different laboratories have not yet been unified, which may lead to inconsistencies in results. Although pNFH shows high specificity in CJD, it may also be elevated in some other rapidly progressive dementias, and the limitations of using pNFH alone for diagnosis are relatively large.

The characteristics of the above emerging biomarkers are summarized in Table 2.

## 7. CSF Biomarkers in Differential Diagnosis of CJD

### 7.1. Differentiation from AD

Differential diagnosis between sCJD and rapidly progressive AD is clinically challenging due to overlapping cognitive decline, myoclonus, and MRI abnormalities. Recent studies have highlighted the role of CSF biomarkers, particularly t-tau and the ratio of amyloid-beta (Aβ) isoforms, in enhancing diagnostic accuracy. Elevated levels of t-tau have been consistently observed in both CJD and AD; however, the t-tau/Aβ42 ratio has emerged as a more specific marker for distinguishing between these two diseases. For instance, a study reported that the t-tau/Aβ42 ratio demonstrated an AUC of 0.992 for differentiating sCJD from rapidly progressive AD, indicating a high diagnostic performance [22]. This biomarker ratio not only aids in the identification of CJD but also helps mitigate false positives that can arise from elevated t-tau levels in AD. Furthermore, the incorporation of additional biomarkers such as p-tau has been suggested to enhance the diagnostic framework. The integration of these biomarkers into clinical practice could lead to more accurate and timely diagnoses, ultimately improving patient management and outcomes.

### 7.2. Distinguishing CJD from Other Rapidly Progressive Dementias (RPD)

In the context of RPD, the differential diagnosis between CJD and conditions such as autoimmune encephalitis is critical yet complex. 14-3-3 protein, traditionally used as a biomarker for CJD, has shown varying diagnostic performance when compared to other emerging biomarkers. A recent study found that while 14-3-3 protein levels were significantly elevated in CJD patients, they also exhibited high levels in certain non-prion conditions, leading to potential misdiagnosis [29]. In contrast, biomarkers such as NfL and brain-derived tau (BD-tau) have shown promise in distinguishing CJD from non-prion RPDs. For example, the plasma BD-tau/p-tau217 ratio has been reported to match the diagnostic accuracy of CSF 14-3-3 in differentiating CJD from non-prion rapidly progressive dementias [11]. This highlights the necessity of using a multimodal biomarker approach to enhance diagnostic precision. The combination of CSF and plasma biomarkers, alongside clinical assessments, may provide a robust framework for accurately identifying CJD amidst other rapidly progressive dementias.

Here is a structured diagnostic approach is recommended: injury screening (t-tau + 14-3-3/NSE/NfL), confirmatory prion testing (CSF RT-QuIC, PrP oligomer assays when available), and differential refinement: Aβ42, p-tau/t-tau ratio, autoimmune/paraneoplastic antibody work-up. This tiered workflow reduces misclassification and supports real-time clinical decision-making. Continued research into the specificity and sensitivity of these biomarkers is essential to refine diagnostic protocols and improve patient care in this challenging clinical landscape.

## 8. Technical Barriers and Requirements for Standardization

### 8.1. Sensitivity/Specificity Constraints in Assay Platforms

The detection of CSF biomarkers is fraught with challenges, particularly concerning the sensitivity and specificity of various assays employed across different laboratories. Variability in results among laboratories can significantly impact the reliability of biomarker detection, which is crucial for accurate diagnosis and monitoring of conditions such as CJD and AD. This variability can arise from differences in sample handling, assay protocols, and the specific antibodies used. For example, the choice of antibodies can influence the sensitivity of the assay, as some antibodies may have higher affinities for their targets than others, leading to discrepancies in biomarker quantification [53]. Furthermore, the detection platforms themselves, whether they are ELISA, mass spectrometry, or other methods, can yield different levels of sensitivity and specificity. The lack of standardized protocols across laboratories can result in significant inter-laboratory variability, which complicates the interpretation of biomarker data and can lead to misdiagnosis or inappropriate treatment decisions. Addressing these challenges necessitates the development of standardized guidelines and protocols that ensure consistency in biomarker detection methodologies, thereby enhancing the reliability of CSF biomarker assays in clinical practice.

### 8.2. International Normalization and Quality Control Standards

International standardization and quality control are essential for the effective implementation of CSF biomarker detection methods. The development and application of international reference materials play a pivotal role in achieving standardization across laboratories. Such reference materials serve as benchmarks for assay performance, allowing laboratories to calibrate their assays and ensure that results are comparable across different settings. The establishment of these materials involves rigorous testing and validation to confirm their stability and relevance to clinical conditions [54]. Additionally, multi-center studies are critical for analyzing data consistency and validating the performance of CSF biomarkers across various populations and settings. These studies can highlight discrepancies in biomarker levels and help identify factors contributing to variability, such as demographic differences or variations in disease progression. Furthermore, implementing robust quality control measures, including regular proficiency testing and adherence to standardized operating procedures, can enhance the reliability of CSF biomarker assays. By fostering collaboration among international research communities and regulatory bodies, it is possible to create a framework that supports the standardization of CSF biomarker detection, ultimately improving diagnostic accuracy and patient outcomes in neurodegenerative diseases.

## 9. Future Research and Biomarker Integration Strategies

### 9.1. Novel Biomarker Discovery and Multi-Omics Validation

The discovery and validation of novel biomarkers for diseases such as CJD are increasingly reliant on advanced multi-omics technologies, particularly proteomics and metabolomics. Proteomics, which involves the large-scale study of proteins, allows for the identification of differentially expressed proteins in CSF samples from CJD patients compared to healthy controls. For instance, recent studies have demonstrated the effectiveness of mass spectrometry-based proteomics in identifying specific protein profiles associated with neurodegenerative diseases, highlighting potential biomarkers that could aid in early diagnosis and monitoring of disease progression [55]. Similarly, metabolomics, which focuses on the comprehensive analysis of metabolites within biological samples, can uncover metabolic disturbances that may serve as indicative biomarkers for CJD. The integration of these two approaches can enhance the specificity and sensitivity of biomarker discovery by providing a more holistic view of the pathophysiological changes occurring in CJD. For example, metabolomic profiling has revealed distinct patterns of metabolites that correlate with disease severity and progression, thus offering insights into the underlying biochemical pathways involved in CJD [56]. The synergistic application of proteomics and metabolomics not only facilitates the identification of novel biomarkers but also enhances the validation process by corroborating findings across multiple biological layers, ultimately leading to more robust diagnostic tools.

### 9.2. Combined Biomarker Panels and Diagnostic Algorithms

In the diagnosis of prion diseases, combining multiple biomarkers has shown promise in improving diagnostic accuracy (Table 3).

Senesi et al. conducted a systematic evaluation of cerebrospinal fluid biomarkers for the diagnosis of sCJD in 50 neuropathologically confirmed patients and 48 non-CJD controls, comprising 15 AD cases and 33 other non-CJD neurological diseases, using ELISA, WB, and RT-QuIC assays. When each modality was analyzed separately, RT-QuIC with recombinant full-length HaPrP (23-231) as substrate demonstrated 92.7% sensitivity and 100% specificity; total tau protein was measured on the automated Roche Elecsys^®^ immunoassay platform (Roche Diagnostics, Basel, Switzerland) with a cutoff of 536 ng/L, yielding 90% sensitivity and 90% specificity; and 14-3-3 protein, assessed by Circul Lex™ ELISA (CircuLex Co., Ltd., Nagano, Japan) with a cutoff of 2.5 ng/mL, showed 81.3% sensitivity and 84.4% specificity, with concurrent validation by WB (sensitivity 87.5%, specificity 66.7%). When employing a three-marker combined strategy, diagnostic sensitivity reached 98% (49/50), with an overall miss rate of 2% (1/50), markedly lower than the miss rate for RT-QuIC alone (7.3%), thereby providing a more reliable screening approach for high-risk populations [1].

The integration of CSF biomarkers with deep learning can further enhance prediction accuracy. The NCJDRSU team from the University of Edinburgh discovered that combining CSF S100B with *PRNP*-129 polymorphism can facilitate risk stratification. Utilizing the national surveillance cohort data from 2017 to 2022, which included 655 confirmed or highly probable sCJD cases, they compared the traditional Cox proportional hazards model with their self-developed deep survival network NMTLR (neural multi-task logistic regression). The data comprised 21 features, such as initial symptoms, MRI/electroencephalogram (EEG) impressions, CSF RT-QuIC, 14-3-3, S100B, and *PRNP*-129 genotype. After temporal 8:2 splitting, tenfold MICE imputation, and bootstrap evaluation, NMTLR achieved a c-index of 0.732, an integrated Brier score (IBS) of 0.079, and an AUC of 0.866 at 5 months, outperforming Cox in calibration and without the need for proportional hazards assumptions. Model-agnostic interpretation revealed that *PRNP*-129 polymorphism contributed the most, followed by 14-3-3 NMTLR automatically captured non-linear interactions, such as age and cortical lesion count, while Cox only exhibited linear relationships. Based on this study, we can construct a “diagnosis-as-prediction” individualized model: clinicians simply need to input the patient’s initial symptoms, *PRNP*-129 genotype, CSF S100B and 14-3-3 results, and MRI/EEG impressions, which are routinely available data, and the system will immediately output an interpretable individualized survival curve. This provides a quantitative basis for prognosis communication, care arrangement, and clinical trial stratification, achieving precise and individualized clinical management of sCJD [57].

The integration of CSF biomarkers with imaging techniques and blood biomarkers presents a promising avenue for enhancing diagnostic accuracy and patient stratification in CJD. Recent advancements in neuroimaging, such as positron emission tomography (PET) and MRI, have demonstrated the capability to visualize pathological changes in the brain associated with neurodegenerative diseases, including CJD. For instance, studies have shown that imaging biomarkers can complement CSF biomarkers by providing spatial and temporal insights into disease progression [58]. The combination of these modalities allows for a more comprehensive assessment of the disease state, as CSF biomarkers can indicate biochemical changes, while imaging can reveal structural and functional alterations in the brain. Furthermore, the addition of blood-based biomarkers, such as NfL and glial fibrillary acidic protein (GFAP), can further enhance diagnostic precision by providing a non-invasive means of monitoring disease progression [59]. The development of multi-modal biomarker panels that incorporate CSF, imaging, and blood biomarkers could lead to improved diagnostic algorithms that enable earlier detection and more effective monitoring of CJD, ultimately guiding therapeutic interventions and improving patient outcomes.

### 9.3. Mult-Omics Technologies in CJD Diagnosis

The complexity of multi-omics datasets, which include genomic, transcriptomic, proteomic, and metabolomic information, poses significant challenges for traditional analytical methods. However, machine learning algorithms can effectively manage these high-dimensional data, uncovering patterns and relationships that may not be apparent through conventional analysis [60]. The integration of multi-omics with minimally invasive liquid biopsies holds promise for enabling early detection of CJD. For instance, beyond CSF, non-invasive sources such as saliva, blood, or urine can be analyzed for metabolomic, proteomic, and RNA expression profiles to identify CJD-associated biomarkers [19], thereby facilitating the development of early diagnostic tools.

Multi-omics technologies also offer potential for constructing risk prediction models for CJD. By integrating genomic data with gut microbiome and metabolomic profiles, it becomes possible to identify environmental and genetic factors associated with CJD pathogenesis [61], paving the way for personalized therapeutic strategies. Furthermore, the application of machine learning algorithms can enhance the performance of such multi-omics-based predictive models. Through feature selection and pattern recognition in high-dimensional datasets, machine learning approaches can significantly improve the accuracy and robustness of CJD risk prediction.

### 9.4. Artificial Intelligence-Integrated Multi-Modal Systems

Artificial intelligence (AI), particularly machine learning, has emerged as a powerful tool for integrating and analyzing multi-omics data in the context of CJD diagnosis. Advancements in machine learning, particularly genetic algorithms, offer promising methodologies for analyzing complex biomedical datasets. These approaches have the potential to facilitate timely and accurate diagnosis, thereby improving clinical outcomes [62]. By examining CFS or blood samples, machine learning algorithms can identify abnormal accumulations of prion proteins. This approach holds promise for developing less invasive diagnostic tests [62]. Except that, Machine learning models also can analyze genetic data to identify mutations or variants linked to heightened risk for prion diseases. Early identification through this approach can inform personalized monitoring and treatment plans [62].

In summary, machine learning models can be trained to recognize specific biomarker signatures associated with CJD by analyzing integrated datasets that combine CSF proteomics, metabolomics, and clinical data. This integrative approach not only enhances the predictive accuracy of diagnostic models but also facilitates the identification of novel biomarkers that could serve as therapeutic targets. Furthermore, AI-driven models can continuously learn and adapt as new data becomes available, improving their performance over time. The application of machine learning in the analysis of multi-omics data holds great promise for advancing the understanding of CJD pathophysiology and developing more effective diagnostic and therapeutic strategies, ultimately paving the way for personalized medicine in neurodegenerative diseases [63].

## 10. Conclusions

**Figure 1:**
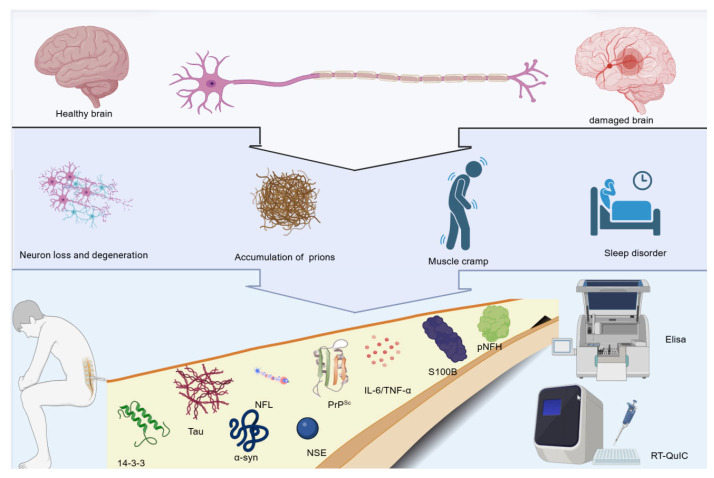
Overview of CSF biomarkers in CJD. Figure illustrates the CSF biomarkers in CJD. The upper part of the figure presents a comparison between a healthy brain and a damaged brain. When the brain is damaged, pathological changes such as neuron loss and degeneration, and the accumulation of prions occur, leading to symptoms such as muscle cramps and sleep disorders. The lower part of the figure lists various CSF biomarkers related to CJD, including 14-3-3, Tau, α-synuclein, NSE, PrP^Sc^, IL-6/TNF-α, S100B, pNFH, and NFL. Laboratory techniques for detecting these biomarkers, such as Elisa and RT-QuIC are shown, which are helpful for the diagnosis and research of CJD.

CSF biomarkers have transformed the diagnostic landscape of CJD, enabling pre-mortem identification and improved differential accuracy among rapidly progressive dementias. Currently, a large number of biomarkers for CJD have been researched and applied, as shown in Figure 1. Among the existing CSF biomarkers, RT-QuIC remains the most specific and reliable assay for prion-seeding confirmation, while surrogate markers such as t-tau, NfL, pNFH, and α-synuclein quantify neurodegeneration and support prognostic estimation. Yet substantial challenges persist, including inter-laboratory heterogeneity, incomplete assay standardization, and limited early-stage sensitivity. Future progress will depend on harmonized CSF handling workflows, externally validated biomarker cut-offs, and integration of multi-omics screens with machine-learning diagnostic platforms. A multi-tier biomarker model—combining prion-specific amplification, quantitative injury indicators, and AI-driven subtype classifiers—offers a realistic path toward earlier detection, precision therapy design, and improved patient outcome prediction.

## Figures and Tables

**Table 1:** Diagnostic performance summary of core CSF biomarkers in CJD.

Biomarker	Detection Method	Advantages	Limitations	Clinical Utility
14-3-3	ELISA or automated immunoassay (Western blot used mainly for historical or experimental reference)	High sensitivity (70–85%) in sporadic Creutzfeldt–Jakob Disease (sCJD); widely used; supports rapid differential diagnosis	Low specificity; elevated in other dementias (e.g., Frontotemporal Dementia)	Supportive biomarker, best used in panels
Tau (t-tau and p-tau)	ELISA or automated immunoassay (Western blot used mainly for historical or experimental reference)	High diagnostic accuracy vs. rapidly progressive AD (AUC 0.94); reflects synaptic injury and neuronal loss	Subtype-dependent variability; p-tau alone less informative	Useful for distinguishing CJD from AD; ratio analysis improves specificity
PrP^Sc^	RT-QuIC	Very high sensitivity (90–95%) and specificity (>95%); rapid and reproducible; potential for early diagnosis	Requires assay standardization; inter-laboratory variability	Current gold-standard CSF test for sCJD

Note: Values reflect representative multi-center cohorts; assay cut-offs vary by laboratory and should not be interpreted in isolation.

**Table 2:** Secondary and Emerging CSF Biomarkers for CJD: advantages, diagnostic utility and limitations.

Biomarker	Detection Method	Advantages	Limitations	Clinical Utility
Neurofilament Light Chain (NfL)	WB/ELISA	Early and sensitive marker of axonal injury; measurable in CSF/serum; prognostic for disease course	Reduced specificity due to overlap with other neurodegenerative diseases	Promising for early diagnosis and progression monitoring
IL-6/TNF-α	ELISA	Reflect neuroinflammation; correlate with severity and progression	Low specificity; confounded by other neuroinflammatory conditions	Adjunct markers for monitoring neuroinflammation
α-synuclein	CSF ELISA/multiplex bead-based immunoassay (Luminex)	Meta-analysis: 92% sensitivity, 96% specificity to separate prion from non-prion disorders; markedly higher in sCJD-MM1 subtype; inverse correlation with survival	Overall diagnostic power still below t-tau/14-3-3; susceptible to blood contamination and platelet release	Subtype classification and prognostic assessment; improves accuracy when combined with tau/14-3-3
S100B	CSF/serum ELISA	Sensitivity comparable to, or higher than, tau/14-3-3 in some cohorts; significantly elevated in gCJD (e.g., E146G) even when 14-3-3 and RT-QuIC are negative; correlates with PRNP codon-129 status, age, disease duration	Limited specificity—also rises after stroke, TBI, epilepsy, gliosis; reference ranges not harmonized	Useful for differentiating genetic prion disease; negative result helps rule out acute cerebral injury
NSE	CSF ELISA	Sensitive to neuronal injury; increases early; longitudinal studies show continual rise with disease course	Low specificity (~91%); elevated in neuro-endocrine tumours, ischaemia, seizures; at 35 ng/mL cut-off specificity only 92%	Rapid screening adjunct; serial measurements indicate progression speed
pNFH	CSF ELISA or Simoa	AUC > 0.9 for distinguishing CJD from other neurodegenerations; higher sensitivity & specificity than 14-3-3, especially in younger patients	Assay protocols and cut-offs not yet standardized; some rapidly progressive dementias (auto-immune encephalitis, paraneoplastic) also show elevation	Initial diagnosis, treatment-response evaluation, and prognosis prediction; dynamic monitoring of therapeutic interventions

**Table 3:** Advantages of Biomarkers Combination in CJD.

Combinations	Detection Method	Advantages
RT-QuIC, total tau, 14-3-3	RT-QuIC (recombinant HaPrP), Roche Elecsys^®^ immunoassay (tau), ELISA/WB (14-3-3)	98% sensitivity, 2% miss rate (superior to single-marker approaches), reliable for high-risk screening
CSF S100B, *PRNP*-129 polymorphism	NMTLR deep learning model	Enables risk stratification; c-index 0.732, IBS 0.079, AUC 0.866; outputs interpretable survival curves for individualized prognosis and management
CSF, imaging (PET/MRI), blood biomarkers (e.g., NfL, GFAP)	Multi-modal integration (biochemical, structural, functional assays)	Comprehensive disease assessment: combines biochemical, spatial, temporal, and non-invasive monitoring for earlier detection and improved therapeutic guidance

## Data Availability

The original contributions presented in this study are included in the article. Further inquiries can be directed to the corresponding authors.

## Abbreviations

The following abbreviations are used in this manuscript:

Aβ	amyloid-beta
AUC	Area Under Curve
AD	Alzheimer’s Disease
BD-tau	Brain-derived Tau
CJD	Creutzfeldt–Jakob Disease
CNS	Central Nervous System
CSF	Cerebrospinal Fluid
DWI	Diffusion Weighted Imaging
ELISA	Enzyme-Linked Immunosorbent Assay
gCJD	Genetic CJD
GFAP	Glial Fibrillary Acidic Protein
IL-6	Interleukin-6
MRI	Magnetic Resonance Imaging
NfL	Neurofilament Light Chain
pNFH	Phosphorylated Neurofilament Heavy Chain
PD	Parkinson’s Disease PD
PET	Positron Emission Tomography
p-tau	Phosphorylated Tau
PrP	Prion Protein
PrP^C^	Cellular Prion Protein
PrP^Sc^	Pathogenic Prion Protein
RPD	Rapidly Progressive Dementia
RT-QuIC	Real-Time Quaking-Induced Conversion
sCJD	Sporadic Creutzfeldt–Jakob Disease
t-tau	Total Tau
TNF-α	Tumor Necrosis Factor-Alpha
WB	Western Blot
